# Visible gland constantly traces virus-induced gene silencing in cotton

**DOI:** 10.3389/fpls.2022.1020841

**Published:** 2022-09-16

**Authors:** Zhanfeng Si, Huaitong Wu, Yue Tian, Zhiyuan Zhang, Tianzhen Zhang, Yan Hu

**Affiliations:** ^1^Department of Agronomy, College of Agriculture and Biotechnology, Zhejiang University, Hangzhou, China; ^2^Key Laboratory for Tree Breeding and Germplasm Improvement, Southern Modern Forestry Collaborative Innovation Center, College of Forestry, Nanjing Forestry University, Nanjing, China; ^3^College of Biotechnology, Jiangsu University of Science and Technology, Zhenjiang, China; ^4^Hainan Institute of Zhejiang University, Sanya, China

**Keywords:** cotton, gene function analyses, VIGS (virus-induced gene silencing), marker gene, gland trait

## Abstract

A virus-induced gene silencing (VIGS) system was established to induce endogenous target gene silencing by post-transcriptional gene silencing (PTGS), which is a powerful tool for gene function analysis in plants. Compared with stable transgenic plant *via Agrobacterium-mediated* gene transformation, phenotypes after gene knockdown can be obtained rapidly, effectively, and high-throughput through VIGS system. This approach has been successfully applied to explore unknown gene functions involved in plant growth and development, physiological metabolism, and biotic and abiotic stresses in various plants. In this system, *GhCLA1* was used as a general control, however, silencing of this gene leads to leaf albino, wilting, and plant death ultimately. As such, it cannot indicate the efficiency of target gene silencing throughout the whole plant growth period. To address this question, in this study, we developed a novel marker gene, *Gossypium PIGMENT GLAND FORMATION GENE* (*GoPGF*), as the control to trace the efficiency of gene silencing in the infected tissues. *GoPGF* has been proved a key gene in gland forming. Suppression of *GoPGF* does not affect the normal growth and development of cotton. The number of gland altered related to the expression level of *GoPGF* gene. So it is a good marker that be used to trace the whole growth stages of plant. Moreover, we further developed a method of friction inoculation to enhance and extend the efficiency of VIGS, which facilitates the analysis of gene function in both the vegetative stage and reproductive stage. This improved VIGS technology will be a powerful tool for the rapid functional identification of unknown genes in genomes.

## Introduction

As a kind of hereditary immune mechanism prevalent in plants, virus-induced gene silencing (VIGS) belongs to post-transcriptional gene silencing (PTGS) (Lu et al., 2003). When carrying a vector with endogenous gene fragments of the host plant infection, VIGS can activate the plant’s own immune system. Plants recognize and degrade viral RNA, and also produce microRNAs that contain endogenous genes of interest. Through binding to the mRNAs of the target genes, these microRNAs can form a hairpin structure which is then degraded by the Dicer enzyme, leading to a decrease in the expression level of the gene of interest or loss of function (Senthil-Kumar and Mysore, 2011).

As one of the most widely used gene silencing systems, the tobacco rattle virus-induced gene silencing system (TRV-VIGS) has the advantages of high silencing efficiency, long duration, light symptoms of the host plant viruses, and no cover of the phenotype in various organizations that produce gene silencing. Above all, as it does not require the development of stable transformants, it is very suitable for characterizing gene function in plants with low transformation efficiency. Therefore, due to its validity and rapidity, TRV-VIGS has been successfully used in various plants, such as *Nicotiana tabacum*, *Solanum lycopersicum*, *Gossypium hirsutum*, *Arabidopsis*, *Capsicum annuum*, etc. (Tian et al., 2014).

**FIGURE 1:**
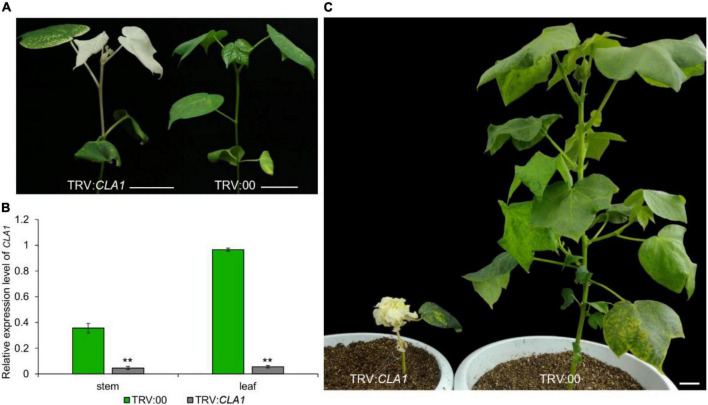
The gene expression and phenotype of the *CLA1*- silenced plants. **(A)** After 2 weeks of silencing the *CLA1* gene, the leaves showed an albino phenotype. **(B)** After 2 weeks of silencing the *CLA1* gene, the expression of genes in young leaves and young stems of albino and empty control plants were analyzed. **(C)** The phenotype of the plant was observed after silencing the *CLA1* gene for 2 months. Error bars are s.d. of three biological repeats. **P < 0.01; Student’s *t*-test, *n* = 3. Bars = 5 cm.

The VIGS method utilizes the *Cloroplastos alterados 1* gene (*CLA1*) as a common positive control, which results in photobleaching of the silenced regions (Gao et al., 2011a). Recently, VIGS technology has been used extensively to study the gene function of cotton, due to its discrete characters verification, such as Red plant (*R*_1_) (Cai et al., 2014), Okra leaf (*L_2_^0^*) (Chang et al., 2016; Andres et al., 2017), Glandless (*Gl_2_*^e^**) (Ma et al., 2016), Ligon-lintless (*Li*_1_) (Thyssen et al., 2017), Naked seed (*N*_1_) (Wan et al., 2016), Lint fiber development (*Li*_3_) (Wu et al., 2018), Axillary flowering (*GbAF*) (Si et al., 2018), Coiled-coil nucleotide-binding site leucine-rich repeat (*CC-NBS-LRR*) (Deng et al., 2019), APETALA 2/ethylene-responsive factor (*AP2/ERF*) (Pei et al., 2021), UBX-Domain Containing10 (Zang et al., 2021a) and novel stem pigment gland-forming gene (*GoSPGF*) (Zang et al., 2021b). There are many studies on the analysis of gene function in biotic and abiotic resistances including disease (Gao et al., 2011b), drought (Chen et al., 2015) and low temperature (Liu et al., 2015). However, after the whitening of the individual plants, the treated plants grow slowly and weakly compared to normal plants, due to their inability to complete the photosynthesis process. Finally, after the albino plant grows for about 2 months, it gradually withers (Figure 1). Therefore, this positive control is suitable only for identifying the phenotype at the seedling stage, and is not conducive to the study of the phenotype at the middle or late growth stage. Here, we report on a novel positive control gene *GoPGF* (Ma et al., 2016), which could constantly trace virus-induced gene silencing visually throughout the entire range of plant growth stages. Thus, it is suitable for studying traits such as buds, flowering, and fiber.

## Materials and methods

### Plant materials

The materials used in the experiments were TM-1 with glands. TM-1 is a standard genetic line of Upland cotton and has broad leaves. The seeds were germinated and grown in a greenhouse. The seedlings from the cotyledon expansion to 4th true leaf emergence were used for the VIGS assays. Infiltrated plants were grown in a growth chamber at 23°C with a 16/8 h light/dark photoperiod. *N. benthamiana* was grown at 25°C in a semi-controlled walk-in chamber with a 16/8 h light to dark photoperiod for the generation of transgenic plants or agroinfiltration.

### Recombinant virus-induced gene silencing vectors

The pTRV1, pTRV-*CLA1*, and pTRV-*GoPGF* vectors were stored in our laboratory. The three vectors were transformed into *Agrobacterium tumefaciens* strains GV3101.

### *Agrobacterium* infiltration

pTRV1, pTRV-*CLA1*, and pTRV-*GoPGF* were grown overnight at 28°C in an antibiotic selection medium containing rifampicin and kanamycin at a concentration of 50 mg l^–1^. *Agrobacterium* cells were collected and resuspended in an infiltration medium (10 mM MgCl_2_, 10 mM MES and 200 μM acetosyringone), and adjusted OD_600_ to OD_600_ 1.5. When silencing *GoPGF* gene separately *Agrobacterium* strains to contain the TRV1 and TRV-*GoPGF* vectors were mixed at a ratio of 1:1. While silencing the *CLA1* and *GoPGF* genes at the same time, in the case of the *Agrobacterium* strains carrying pTRV-*CLA1*, pTRV- *GoPGF*, and pTRV1 vectors were mixed at a ratio of 1: 1: 2 and then injected into the cotton cotyledons. The treated cotton seedlings were grown in a growth chamber at 23°C with a 16/8 h light/dark photoperiod.

### Friction inoculation in the virus-induced gene silencing system

The tobacco (*N. benthamiana*) grows to 6 leaves in a greenhouse at 25°C in a semi-controlled walk-in chamber with a 16/8 h light to dark photoperiod. TRV1 and TRV-*CLA1* were grown overnight at 28°C in a LB medium containing antibiotics (50 μg/ml kanamycin, 25 μg/ml gentamicin), 10 mM MES and 20 μM acetosyringone. The cells were pelleted by centrifugation at 1,200 × *g* at room temperature for 8 min, and resuspended in an MMA (10 mM MES, 10 mM MgCl2, 200 μM acetosyringone) solution to the final OD_600_ of 1.5. Cell suspensions were incubated at room temperature for at least 3 h without shaking. The *Agrobacterium* strains containing the TRV1 and pTRV-*CLA1* vectors were mixed at a ratio of 1:1 and infiltrated into tobacco leaves using a needleless syringe. The treated tobacco was grown in a greenhouse at 23°C with a 16/8 h light/dark photoperiod.

Tobacco was placed in the greenhouse after 2 weeks of growth, with the new leaves showing white spots. In the mortar, white-spotted tobacco leaves were added, along with a little 400 mesh quartz sand and 10 ml 0.01M phosphate buffer solution (PBS). These were then ground into a homogenate.

In the cotton plant, only the completely unfolded leaves were chosen, and a small quantity of 400 mesh quartz sand was sprinkled over the above mixture. A pestle was used to divert the prepared homogenate to gently rub over the selected cotton leaves. Friction control was implemented in order to penetrate the leaves, so that the leaves would be damaged to the epidermis. The purpose was intended to invade cotton leaves with the tobacco containing virus. The treated cotton was placed in a greenhouse at 23°C with a 16/8 h light/dark photoperiod. After 2–3 weeks, the new growing cotton leaves displayed whitening phenotypes.

### RNA extraction and quantitative real-time PCR analysis

RNA was extracted from the stem, leaf, bract, and calyculus of TM-1 using the Biospin Plant Total RNA Extraction Kit (Hangzhou Bori Technology Co. Ltd, Hangzhou, China, cat: BSC65S1). First-strand cDNA was generated using a TransScript^®^ One-Step gDNA Removal Kit and a cDNA Synthesis SuperMix (TransGen Biotech Co. Ltd, Beijing China, cat: AT311) according to the manufacturer’s instructions. The cotton *Histone 3* gene (AF024716) was used as the housekeeping gene. The primers’ sequences were listed in Supplementary Table 1.

## Results

### The number of glands on the plants reflects the silencing efficiency of virus-induced gene silencing

**FIGURE 2:**
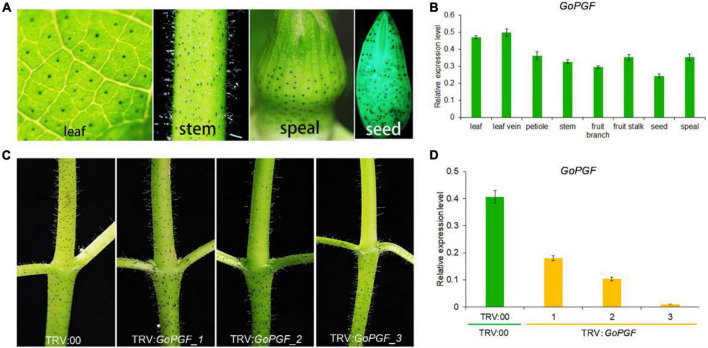
Gland phenotypes and gene expression level in different tissues of *GoPGF*-silenced plants. **(A)** Distribution of glandular phenotype in different tissues of cotton including leaf, stem, bud and seed. **(B)** Relative expression of the *GoPGF* gene in different tissues of cotton. Error bars are s.d. of three biological repeats. **(C)** Comparison of silencing efficiency in different VIGS plants. **(D)** The relative expression of the *GoPGF* gene was analyzed in the stems of different VIGS plants in panel **(C)**. Error bars are s.d. of three biological repeats.

The *Gossypium* species are characterized by the presence of small and darkly pigmented lysigenous glands on the surface of almost all the organs (Figure 2A and Supplementary Figure 1). Using a dominant glandless mutant Hai-1, we cloned the pigment gland formation regulator *GoPGF* which encodes a *basic helix-loop-helix* (*bHLH*) transcription factor (Ma et al., 2016). It is expressed in a constitutive manner in most organs and tissues including the stem, leaf, bract, etc. (Figure 2B), consistent with the presence of glands (Ma et al., 2016). *GoPGF* was proven to be specific to gland development and had no noticeable effect on plant growth and development (Gao et al., 2022). The silencing of *GoPGF* can lead to a complete glandless phenotype. However, we observed that there was a noticeable difference in the silencing efficiency in different VIGS-treated plants even though this applied to the same receptor material and was implemented with the same silencing vector injection. For example, a VIGS-silenced *GoPGF* plant displayed different gland phenotypes using the cotton genetic standard line TM-1 as the receptor. Some *GoPGF*-silenced plants did not develop glands at all, suggesting that *GoPGF* is completely silenced, while some plants showed reduced glands (Figure 2C). The relative expression of *GoPGF* gene is also down-regulated with a decrease in the number of glands (Figure 2D). Thus, the *GoPGF* gene can be utilized as a positive control and internal indicator to trace the silencing efficiency of the target gene through phenotype observation of the absence or presence of the gland and variation of gland numbers throughout the entire VIGS stage.

### Assessment of degree and specificity of virus-induced endogenous *GoPGF* gene silencing in cotton

**FIGURE 3:**
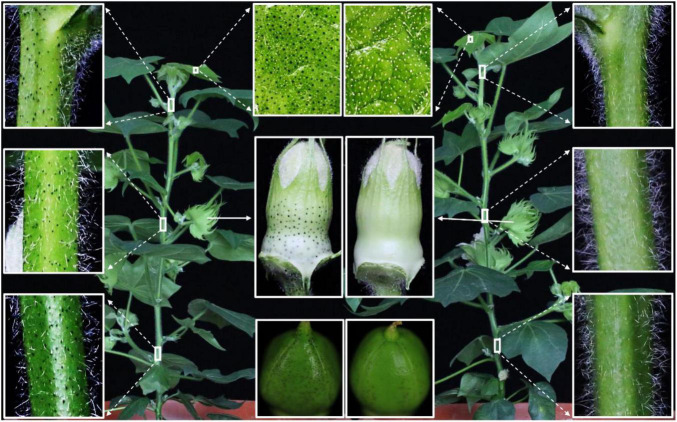
Comparison of the glandular phenotype of different tissues from *GoPGF*-VIGS plants and control at flowering stage. The tissues marked in the white box showed an enlargement of corresponding tissues including leaf, stem, bud and boll from the TRV:00 and TRV:*PGF* whole plants, respectively. The boll was collected 5 days after flowering.

The pTRV-*GoPGF* silencing vector was constructed and injected into TM-1 to observe the glandular phenotype during the completed sequence of developmental stages. Silencing of the *GoPGF* gene resulted in the glandless phenotype in the new leaves and stems after 2–3 weeks of inoculation, while plants inoculated with empty vectors and the control plants displayed a glanded phenotype (Supplementary Figures 2A,B). qPCR analysis revealed that the transcripts of the *GoPGF* gene were significantly decreased in the glandless plants (Supplementary Figure 2). At the flowering stage, some *GoPGF*-silenced plants began to develop a few glands only in the 5-day-old young boll, but no glands were found in the top, middle and lower parts of the stems, the penultimate leaves and the sepals (Figure 3). The expression of the *GoPGF* gene in the six examined tissues was significantly lower than that of the empty vector control (Supplementary Figure 3). Therefore, the silencing efficiency of the endogenous gene can be maintained till the flowering stage. Moreover, when the *GoPGF* gene served as the internal control, the silencing efficiency of the target gene in the plant was directly reflected by the existence and the number variation of glands.

### *GoPGF* can be considered as an internal reference gene in a cotton virus-induced gene silencing system

**FIGURE 4:**
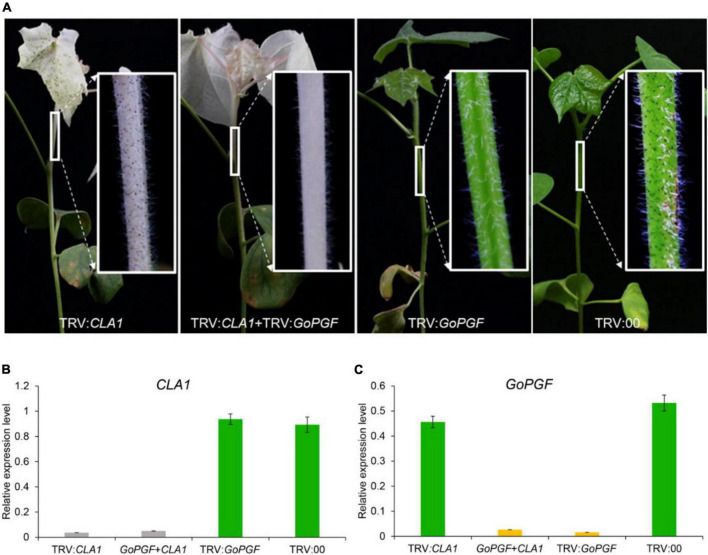
Phenotype and gene expression level of VIGS plants silenced by various combinations of the *CLA1* and *GoPGF* gene. **(A)** Phenotype of VIGS plants silenced by *CLA1* or *GoPGF* gene and both *CLA1*–silenced plants display a typical albino phenotype; the *GoPGF*-silenced plants show a glandless phenotype; VIGS plants showed albino and glandless phenotype when both were silenced simultaneously. **(B)** *CLA1* gene relative expression in the stem marked white box in panel **(A)**. Error bars are s.d. of three biological repeats. **(C)** *GoPGF* gene relative expression in the stem marked white box in panel **(A)**. Error bars are s.d. of three biological repeats.

According to previous reports, two target gene fragments can be constructed on one TRV vector to achieve the purpose of simultaneously silencing two genes (Pang et al., 2013). In this study, a mixed VIGS system was designed by equally mixing *Agrobacterium* containing TRV vectors of different target genes. We utilized the silencing of the *CLA1* gene in order to produce leaf albino and silenced the *GoPGF* gene in order to create glandless traits to validate the above ideas. *Agrobacterium* strains carrying pTRV-*CLA1*, pTRV-*GoPGF* and pTRV1 vectors were mixed at the ratio of 1: 1: 2 and then injected into cotton cotyledons. At the same time, the *Agrobacterium* carrying pTRV-*CLA1* and pTRV-*GoPGF* vectors were, respectively injected into the cotton cotyledons, and the TRV2 empty vector was adopted as the negative control. Two weeks later, the phenotype of the treated plants was observed. The RNA was extracted from the penultimate leaves of cotton plants, and the expression patterns of the *CLA1* and *GoPGF* genes were analyzed. The plants injected with pTRV-*CLA1* had an albino phenotype, while those plants injected with pTRV-*GoPGF* exhibited a glandless phenotype (Figure 4A). Meanwhile, the plants injected with pTRV-*CLA1* mixed with pTRV-*GoPGF* exhibited the white and glandless phenotype simultaneously (Figure 4A). The phenotype of negative control did not change at all (Figure 4A). Expression pattern analysis confirmed that the expression of *CLA1* and *GoPGF* was strongly inhibited when silencing these two genes independently (Figures 4B,C). By silencing pTRV-*CLA1* and pTRV-*GoPGF* at the same time, the expression of both genes was also completely inhibited (Figures 4B,C). Moreover, it is completely feasible to determine the efficiency of the target gene silencing method using the *GoPGF* gene as an internal reference gene by alteration of the gland number.

### Friction inoculation can extend the duration of silencing of target genes

Though the VIGS method has a high silencing efficiency in cotton seedlings, the silencing efficiency after flowering is significantly reduced. Therefore, in order to render this method applicable to the study of gene function in flowering or the fiber elongation stage, we applied a friction inoculation method to extend the duration of silencing of target genes. In addition, the modified method can extend the duration of the VIGS in other plants (Becker and Lange, 2010; Senthil-Kumar and Mysore, 2011). With TM-1 as the receptor material, the *CLA1* gene was used for validating the effectiveness of this approach (Gao et al., 2011a). First, the *Agrobacterium strains* carrying the *CLA1* gene vector were injected into the *Nicotiana tabacum*, and then after 2 weeks, the newly grown tobacco leaf displayed a macular phenotype (Supplementary Figure 4A). Next, 1–3 g of tobacco leaves with the macular phenotype were collected and placed in a mortar. A small amount of PBS (0.01 M, pH = 7.2) was added and ground into juice. The cotton leaves were vaccinated and sprinkled with a trace of 500 mesh diamond. Upon dipping into the juice with a pestle and gently rubbing on the leaves, the leaves exhibited micro-wounds, but epidermal cells were not destroyed. Finally, the treated cotton plants were further grown in a greenhouse. After 2 weeks, the new cotton leaves grew albino (Supplementary Figure 4B). The relative expression of the *CLA1* gene was analyzed by the whitening and normal leaves in the same position of the cotton plants. The expression of the *CLA1* gene in the white leaves was significantly decreased (Supplementary Figure 4C). This result revealed that the friction inoculation method could prolong the silence duration of the target gene in the cotton plants.

## Discussion and conclusions

The earliest application of VIGS in cotton was to construct a *CLA1* gene fragment into a TRV vector and then transform it into *Agrobacterium* to inject cotton cotyledons. After 2–3 weeks, the whitening phenotype of the new leaves was observed, and the target gene was highly efficient (Gao et al., 2011a). Since then, many researchers have used this method for functional gene research and used the *CLA1* gene as a positive control. For example, *GhMLP28* is involved in resistance to *Verticillium wilt* (Yang et al., 2015), *LMI1* regulates the development of the okra leaf (Chang et al., 2016; Andres et al., 2017) and *GhSnRK2* participates in the regulation of drought resistance (Bello et al., 2014), etc. In *Gossypium barbadense*, VIGS systems were established using multiple marker genes, including *GaPDS*, *GaCLA1*, *GaANS*, and *GaANR* (Pang et al., 2013). In the above method, although the high silencing efficiency of the target gene in cotton VIGS system was established, the selection of the marker gene is not perfect. These marker genes have certain defects: *CLA1* and *PDS* gene silencing plants have a whitening phenotype, but do not maintain long-term growth; in *GaANS* gene silencing plants, the leaves need to be treated before the phenotype can be observed. Compared with them, as a marker gene, *GoPGF* can well solve the above-mentioned limitations. When the expression of the *GoPGF* gene is silenced, it does not affect the normal growth of the cotton plant, and gene silencing efficiency can be visually observed through the number variation of glands.

In our study, after inoculation of two *Agrobacterium strains* (containing pTRV-*CLA1* and pTRV-*GoPGF* plasmids, respectively) into the same plant, both whitening and glandless phenotypes appeared at the same time. It was demonstrated that mixing two gene-silencing vectors in VIGS can also silence two target genes simultaneously. This result was consistent with previous reports (Gao et al., 2011a). During the VIGS process, the growth environment of the plant determines the silencing efficiency of the target gene. Even though treated with the same material as inoculation receptor, silencing efficiency of target genes still exists individual difference. Therefore, *GoPGF* as an internal reference gene can reflect the silencing efficiency of the target gene in real time according to the number variation of glands in the newly grown tissue of the plant. Although the VIGS system can be applied to the study of the cotton growth period after flowering, the silencing efficiency of its target gene is much lower than that at the seedling stage (Wan et al., 2016). Thus, we developed an improved method of friction inoculation based on the principle that plant viruses can be transmitted between plants. We successfully verified the feasibility of cotton (Wu et al., 2018; Zang et al., 2021a). The method is to inoculate a TRV virus in the bud stage of cotton and to increase the silencing efficiency of the target gene in the newly grown fruit branch by spreading the virus in the cotton plant. Up to now, many genomes of many cotton species have been released and tens of thousands predicated coding genes have been annotated. However, the function of most genes is unknown, and it needs to be proved by experiments. As we all know, gain of function through transgenic expression of the candidate gene in the mutant varieties is the most direct and effective method for functional validation. But it is time consuming and laborious work and sometime difficult to implement because of genotype dependence of genetic transformation in cotton. Obviously, VIGS is a practicable method for quick gene function verification. Here, this improved VIGS method will facilitates the rapid validation of gene function especially during the development of cotton fiber initiation, elongation, lipid metabolism or under biotic and abiotic stresses.

In summary, we employed the *GoPGF* gene as a positive control and an internal reference gene during the VIGS process, so as to reflect the silencing efficiency of the target gene through existence and number variations of glands. The friction inoculation method is a necessary supplement and helps to investigate phenotype at later stages of development in cotton.

## Data availability statement

The original contributions presented in this study are included in the article/Supplementary material, further inquiries can be directed to the corresponding authors.

## Author contributions

ZS, HW, and YT conceived and designed the experiments. YH, ZS, and HW performed the experiments and wrote the manuscript. YT and ZZ participated in the experiments. ZS, HW, YT, TZ, and YH edited the manuscript. All authors read and approved the last version.
