# Supplementary material for: Visible gland constantly traces virus-induced gene silencing in cotton
Source: Front Plant Sci. 2022 Sep 16;13:1020841. doi: 10.3389/fpls.2022.1020841 (PMC9523728; doi:10.3389/fpls.2022.1020841)
Supplement: Supplementary file 1 [file Data_Sheet_3.DOCX]

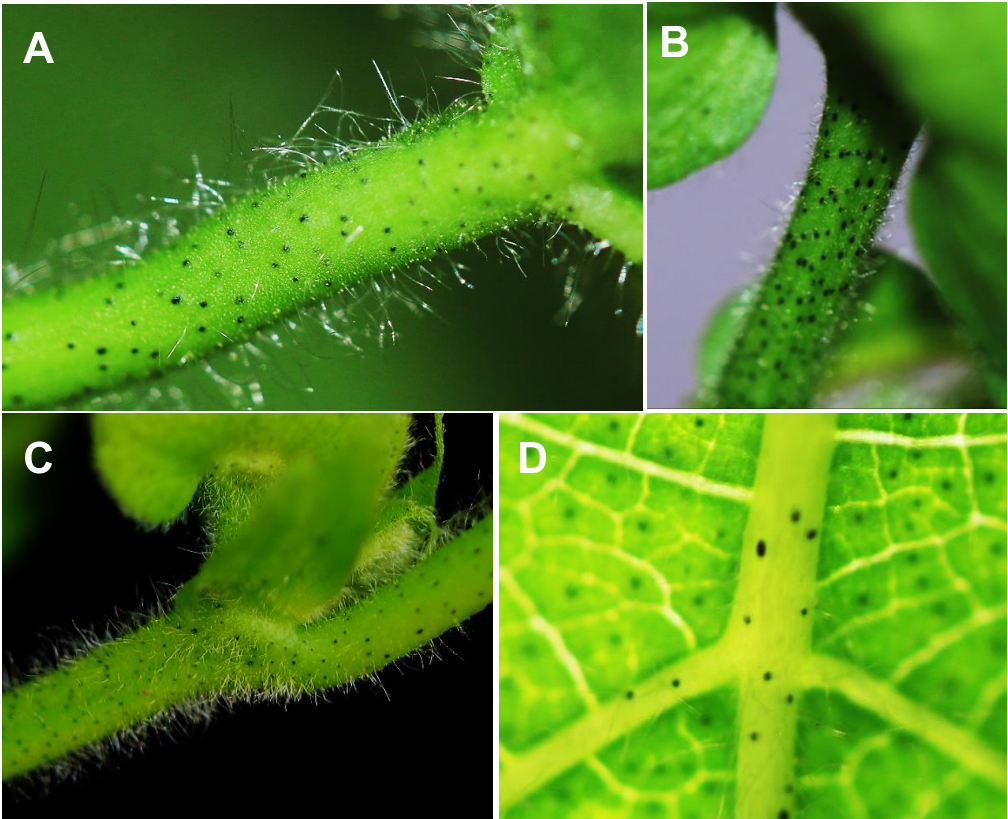


**Fig. S1** Distribution of the glandular phenotype in different tissues of cotton. (A)petiole (B)fruit stalk (C)fruit branch (D)leaf vein.


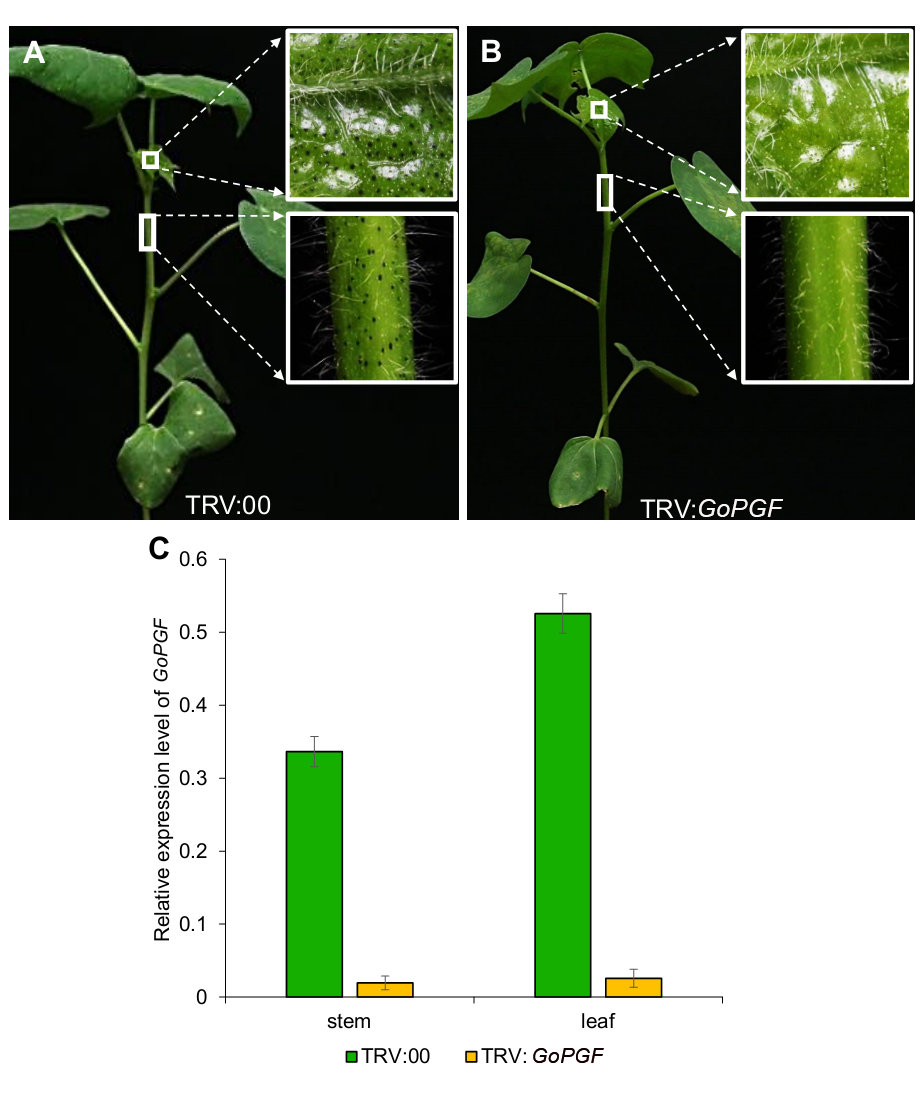


**Fig. S2** In the VIGS system, using the *GoPGF* gene as a positive control for the seedling phenotype observation. (A)Two weeks after the inoculation with the empty vector, the seedling phenotype was observed. (B)Two weeks after the silencing of the *GoPGF* gene, the phenotype was observed at the seedling stage. (C)After 2 weeks of silencing of the *GoPGF* gene, the expression of *GoPGF* was analyzed in the young leaves and stems of the treated and control groups. Error bars are s.d. of three biological repeats. ** P<0.01; Student’s t-test, n=3.


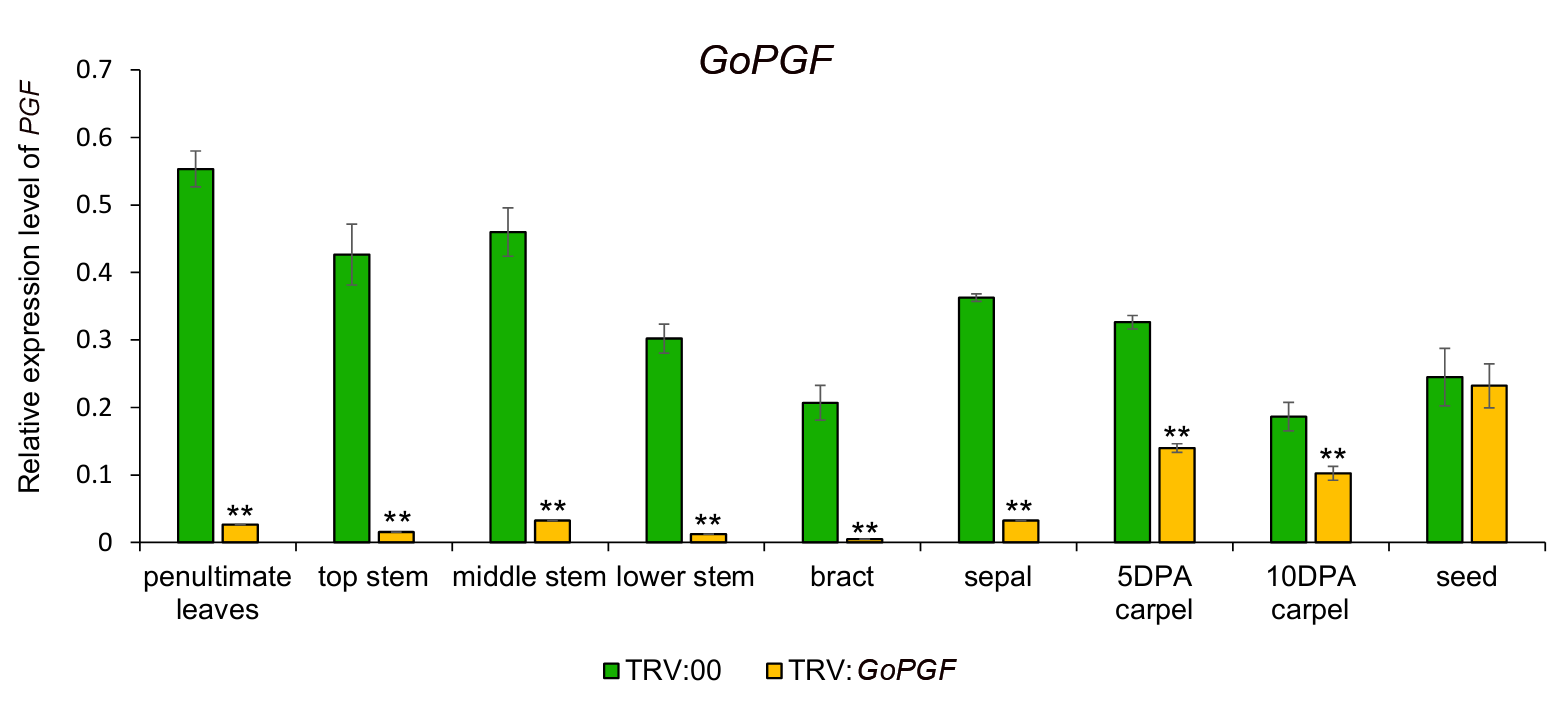


**Fig. S3** The relative expression of *GoPGF* gene was analyzed by selecting the various tissues of silencing *GoPGF* gene and empty vector control. Error bars are s.d. of three biological repeats. ** P<0.01; Student’s t-test, n =3.


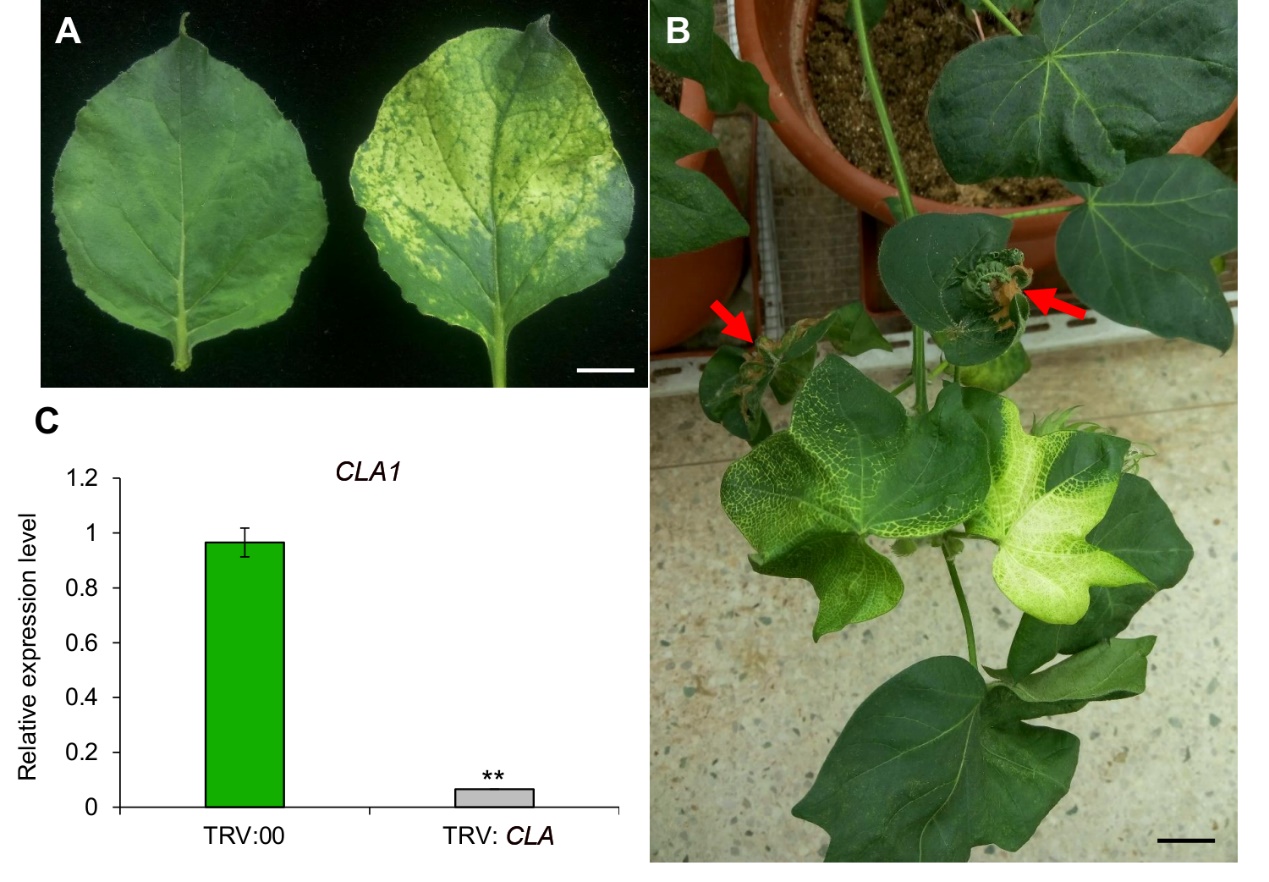


**Fig. S4** Silencing *CLA1* gene was used to verify the effect of friction inoculation. (A)*Agrobacterium tumefaciens* silenced the *CLA1* gene vector into the *Nicotiana tabacum* and the phenotype of the leaves was observed after 2 weeks. (B)The newly developed leaves were inoculated with the leaves of albino tobacco, and the leaf phenotype was observed one month later. The red arrows refer to the leaves that are friction inoculated with leaves. (C)The leaves of albino leaves and the same parts of the corresponding non-rubbing plants were selected to analyze the expression of *CLA1* gene. Error bars are s.d. of three biological repeats. ** P<0.01; Student’s t-test, n =3. Bars=1cm

**Table S1** Quantitative real-time PCR (qPCR) primers used in this study

| Genes | Forward | Reverse |
| --- | --- | --- |
| *GoPGF* | CCCAAGTATTCCTTTTGTTG | CCCTGGTTTTCTACCTCTCT |
| *CLA1* | GTAAAGTGGAGGCCATTGG | AAGCACATTGAACACCGTTG |
| *Histone-3* | CGGTGGTGTGAAGAAGCCTCAT | AATTTCACGAACAAGCCTCTGGAA |
